# Supplementary material for: Deletion of wfs1 Impairs Oligodendrocyte Precursor Cells Dorsal Distribution and Myelination Through the wfs1-hmgcs1 Axis in Zebrafish
Source: Biology (Basel). 2026 Aug 16;15(16):1402. doi: 10.3390/biology15161402 (PMC13510151; doi:10.3390/biology15161402)
Supplement: Supplementary file 1 [file biology-15-01402-s001.zip › File S1-Transcriptome sequencing data and differentially expressed genes (DEGs) in wfs1-- zebrafish at 5 dpf/2.KEGG/wfs1KOvsWT/UP/src/dre04512.html]

dre04512


- dre:321243

- Up regulated genes

321243\_1(9.00901912775666)

- dre:321243

- Up regulated genes

321243\_1(9.00901912775666)

- dre:321243

- Up regulated genes

321243\_1(9.00901912775666)

- dre:321243

- Up regulated genes

321243\_1(9.00901912775666)

- dre:321243

- Up regulated genes

321243\_1(9.00901912775666)

- dre:321243

- Up regulated genes

321243\_1(9.00901912775666)

- dre:321243

- Up regulated genes

321243\_1(9.00901912775666)

- dre:321243

- Up regulated genes

321243\_1(9.00901912775666)

- dre:321243

- Up regulated genes

321243\_1(9.00901912775666)

- dre:321243

- Up regulated genes

321243\_1(9.00901912775666)

- dre:30037

- Up regulated genes

30037(1.55750167372461)

- dre:798998

- Up regulated genes

798998(2.3039125548115)

- dre:30037

- Up regulated genes

30037(1.55750167372461)

- dre:30037

- Up regulated genes

30037(1.55750167372461)

- dre:30037

- Up regulated genes

30037(1.55750167372461)

- dre:100005469

- Up regulated genes

100005469\_1(3.66353583031119)

- dre:100005469

- Up regulated genes

100005469\_1(3.66353583031119)

- dre:100005469

- Up regulated genes

100005469\_1(3.66353583031119)

- dre:100005469

- Up regulated genes

100005469\_1(3.66353583031119)

- dre:100005469

- Up regulated genes

100005469\_1(3.66353583031119)

- dre:100005469

- Up regulated genes

100005469\_1(3.66353583031119)

- dre:100005469

- Up regulated genes

100005469\_1(3.66353583031119)

- dre:100005469

- Up regulated genes

100005469\_1(3.66353583031119)

- dre:100005469

- Up regulated genes

100005469\_1(3.66353583031119)

- dre:100005469

- Up regulated genes

100005469\_1(3.66353583031119)

- dre:100005469

- Up regulated genes

100005469\_1(3.66353583031119)

- dre:564266

- Up regulated genes

564266(2.42311476837666)

- dre:322012

- Up regulated genes

322012\_1(5.87075972359252)

- dre:564266

- Up regulated genes

564266(2.42311476837666)

- dre:570868

- Up regulated genes

570868\_1(5.31304404972279)

- dre:30037

- Up regulated genes

30037(1.55750167372461)

- dre:100005469

- Up regulated genes

100005469\_1(3.66353583031119)

- dre:321243

- Up regulated genes

321243\_1(9.00901912775666)

- dre:563428

- Up regulated genes

563428\_1(1.73680673793125)

Close
